# A Glance at the Use of Glucocorticoids in Rare Inflammatory and Autoimmune Diseases: Still an Indispensable Pharmacological Tool?

**DOI:** 10.3389/fimmu.2020.613435

**Published:** 2021-01-21

**Authors:** Simona Ronchetti, Emira Ayroldi, Erika Ricci, Marco Gentili, Graziella Migliorati, Carlo Riccardi

**Affiliations:** Pharmacology Division, Department of Medicine and Surgery, University of Perugia, Perugia, Italy

**Keywords:** glucocorticoids, rare disease, glucocorticoid-induced leucine zipper, inflammation, autoimmunity

## Abstract

Since their discovery, glucocorticoids (GCs) have been used to treat almost all autoimmune and chronic inflammatory diseases, as well as allergies and some forms of malignancies, because of their immunosuppressive and anti-inflammatory effects. Although GCs provide only symptomatic relief and do not eliminate the cause of the pathology, in the majority of treatments, GCs frequently cannot be replaced by other classes of drugs. Consequently, long-term treatments cause adverse effects that may, in turn, lead to new pathologies that sometimes require the withdrawal of GC therapy. Therefore, thus far, researchers have focused their efforts on molecules that have the same efficacy as that of GCs but cause fewer adverse effects. To this end, some GC-induced proteins, such as glucocorticoid-induced leucine zipper (GILZ), have been used as drugs in mouse models of inflammatory pathologies. In this review, we focus on some important but rare autoimmune and chronic inflammatory diseases for which the biomedical research investment in new therapies is less likely. Additionally, we critically evaluate the possibility of treating such diseases with other drugs, either GC-related or unrelated.

## Introduction

Reading a scientific article that dates back to the year 1900 definitely impresses on how pioneering in unknown medical and biological fields could have been more thrilling than a new discovery in current times, especially because empirical attempts based on experience and medical practice led to unprecedented successes in treating debilitating and life-threatening diseases. For example, the paroxysms of asthma were successfully treated by Doctor Solomon Solis-Cohen by using adrenal extracts administered as “grains of the desiccated gland substance.” It not only provided the suffering patients relief from the recurrence of paroxysms but also helped them reach “a state of freedom from fear of their recurrence.” Such a report clearly describes how debilitating, both in the body and mind, was a disease such as asthma or other chronic inflammatory or autoimmune diseases for which no specific treatment was available. At that time, scientists and physicians were not yet aware of the content of the adrenal extracts, whose benefits were clear, because adrenaline, supposed to be the active compound, was not absorbed when given orally. It took another three decades for the isolation of cortisone and other steroids, which were the active compounds in the treatment of asthma (1). Starting from observations in 1929 and thereafter, Philip Hench, a clinician from the Mayo Clinic, successfully treated patients with rheumatoid arthritis (RA) by administering the so-called “compound E” in 1948. This compound was among the several steroids isolated from the adrenal cortex by the chemist Edward Kendall. Kendall, together with Philip Hench and the biochemist Tadeus Reichstein, was awarded the Nobel Prize in Physiology and Medicine in 1950 for “discoveries related to the hormones of the adrenal cortex, their structure and biological effects.” Compound E was later identified as cortisone, the commercial production of which started in 1949 (2). Its undeniable usefulness in therapy has enabled the treatment of severe pathologies, ranging from autoimmune diseases to tumors, and driven the development of synthetically derived steroid compounds with stronger anti-inflammatory properties. Unfortunately, long-term treatment with glucocorticoids (GCs) is associated with many severe adverse effects, which counteract their beneficial effects (3, 4). To overcome these problems, several attempts have been made thus far to search for new drugs or modified molecules, such as the so-called “dissociated steroids” (5). However, these attempts have been unsuccessful, and seventy years after their first use are still not enough to get rid of these indispensable therapeutics, despite their unwanted adverse effects. Moreover, recently, GCs have been found to be efficacious as life-saving drugs in patients infected with severe acute respiratory syndrome coronavirus 2 (SARS-CoV-2) (6, 7).

## Pleiotropic Aspects of GCs as Anti-Inflammatory Drugs

GCs are, by far, the most effective anti-inflammatory drugs for treating chronic inflammatory diseases, allergies, and autoimmune pathologies, such as RA, asthma, multiple sclerosis, and systemic lupus erythematosus (8, 9). Their mechanism of action has not yet been completely elucidated, owing to the different levels of tissue expression, several glucocorticoid receptor (GR) isoforms, and the complex gene regulation mediated by the activated monomeric or dimeric forms of GR. GC ligands, once bound to the GR, induce series of effects that depend either on the gene regulation activated by the interaction of ligand-bound GR with responsive DNA sequences (genomic actions) and/or molecular events that do not require gene modulation (non-genomic actions) (for a detailed description of the mechanism of action, please refer to references (10, 11)). As drugs, GCs are generally considered immunomodulatory rather than simply immunosuppressive, because of their complex effects on the cells of the immune system. Their anti-inflammatory action is mainly dependent on the suppression of pro-inflammatory cytokines and their transcription factors such as NF-κB and AP-1, and the activation of anti-inflammatory genes, such as *GILZ* (*TSC22D3*) and *DUSP-1* (12–15). Nevertheless, some pro-inflammatory effects of GCs have been reported, such as the induction of the expression of NLRP3, a central component of the inflammasome (16, 17). Furthermore, an extensive body of evidence suggests that GCs have different effects on the immune system depending on the duration of their administration. Prolonged exposure to GCs may cause immunosuppression, whereas acute exposure can activate the immune system (18).

In the case of chronic autoimmune or inflammatory diseases, long-term therapy with high-dose GCs elicits immunosuppressive and anti-inflammatory effects, which are necessary for symptomatic relief. Unfortunately, the consequent adverse effects are sometimes quite severe, requiring specific additional therapies or suspension of GC therapy. In some pathologies, such as asthma, the side effects of GCs have been partially resolved by topical administration (19). However, generally, adverse effects due to high doses cannot be fully avoided in systemic GC therapy. Attempts have been made to develop the so-called dissociated steroids, with the aim to favor the transrepression of activated monomeric GR over GR dimer transactivation, which is considered the cause of side effects. Studies in GR^Dim^ mutant mice, which harbor a mutation that causes impaired homodimerization of the ligand-bound GR, have initially shown a reduced functionality of the transcriptional activity (20). However, other recent studies have revealed that this mutant GR can still dimerize, although to a lower degree, making this model suboptimal for distinguishing differences between GR monomeric and homodimeric related effects (21). Furthermore, some side effects, but also some therapeutic effects, depend on both transactivation and transrepression. Thus, the concept of separating the beneficial anti-inflammatory effects from the adverse effects of GCs cannot be based on the simple separation of transrepression from transactivation activities (10). The dynamics of gene regulation by GR and its binding to the DNA remains a complex mechanism that needs to be more deeply studied. This is the reason why none of the selective glucocorticoid receptor agonist and modulators developed so far has still reached the market. Knowing the biology of the complex functions of these hormones will allow the development of pharmacological tools specifically targeting one of their sophisticated mechanisms (22, 23).

Another important aspect to consider in long-term treatments with GCs is that patients could develop adrenal insufficiency (e.g., 37% of RA patients), because GCs regulate their own secretion through a negative feedback loop, thereby inhibiting the hypothalamic–pituitary–adrenal (HPA) axis (24). It takes some time for the HPA axis to function properly after suppression. Recent studies have demonstrated that it takes as long as 1 year for a suppressed adrenal to again secrete these hormones. Conversely, if GC treatment lasts 1–2 weeks, it takes only 1 day to again secrete endogenous GCs (25). Interestingly, the suppression occurs also locally, at the level of adrenal steroidogenic activity (26). Furthermore, enzymes of the steroid biosynthesis are expressed not only by the adrenal cortex but also by other tissues, such as the lung, brain, spleen, skin and cells of the immune system. Interestingly, dysregulation of local steroidal activity has been found to be involved in the pathogenesis of some autoimmune or inflammatory diseases, such as lupus erythematosus, multiple sclerosis, RA, and psoriasis (27). When local hormone production is altered, many GC-responsive genes are aberrantly expressed and may contribute to the pathogenesis of the above-mentioned diseases.

Sometimes, GC treatments are not efficacious because of the development of resistance to GC effects. Resistance was described first in the 1970s in *in vitro* cell systems and has been largely studied in asthma and RA (28). The lack of a therapeutic response in 4%–10% and 30% of patients with asthma and RA, respectively, is attributable to treatment resistance. Some other inflammatory diseases, such as chronic obstructive pulmonary disease (COPD), are up to 100% resistant to GC treatments (29). There are multiple underlying mechanisms for GC resistance, from those genetic in origin to molecular alterations including the overexpression of the non-ligand–binding GRβ isoform, which functions as a decoy receptor (8, 29, 30). For comprehensive reviews on this subject, see references (31–33). Overcoming the problem of resistance by reactivating the sensitivity to GCs, when possible, is the only strategy for using GC treatment in pathologies that cannot be treated with alternative drugs.

GCs are released by the HPA axis under a specific rhythm, which is regulated by the circadian clock in anticipation of daily energy-demanding situations. In rodents, GC release peaks slightly before nighttime, whereas in humans, this happens before daybreak, just in the proximity of their respective active phase (34). Therefore, the time of administration of exogenous GCs in chronic autoimmune or inflammatory diseases is critical and should be carefully chosen because of the circadian clock. Furthermore, the existence of cross-talk between the HPA axis and the immune system further complicates the scenario in which GCs must act. It has been suggested that late-night administration is more effective than early morning administration, since the immune system starts to be activated between 1:00 and 2:00 a.m. and peaks early in the morning. This immune activation increases in some inflammatory conditions, such as RA, gout, or allergic asthma, and cannot be controlled by endogenous GCs, whose production is already inadequate due to chronic inflammation and consequent downregulation of the HPA axis. Therefore, administration of exogenous GCs during nighttime ameliorates morning symptoms in RA patients (35).

**Figure 1:**
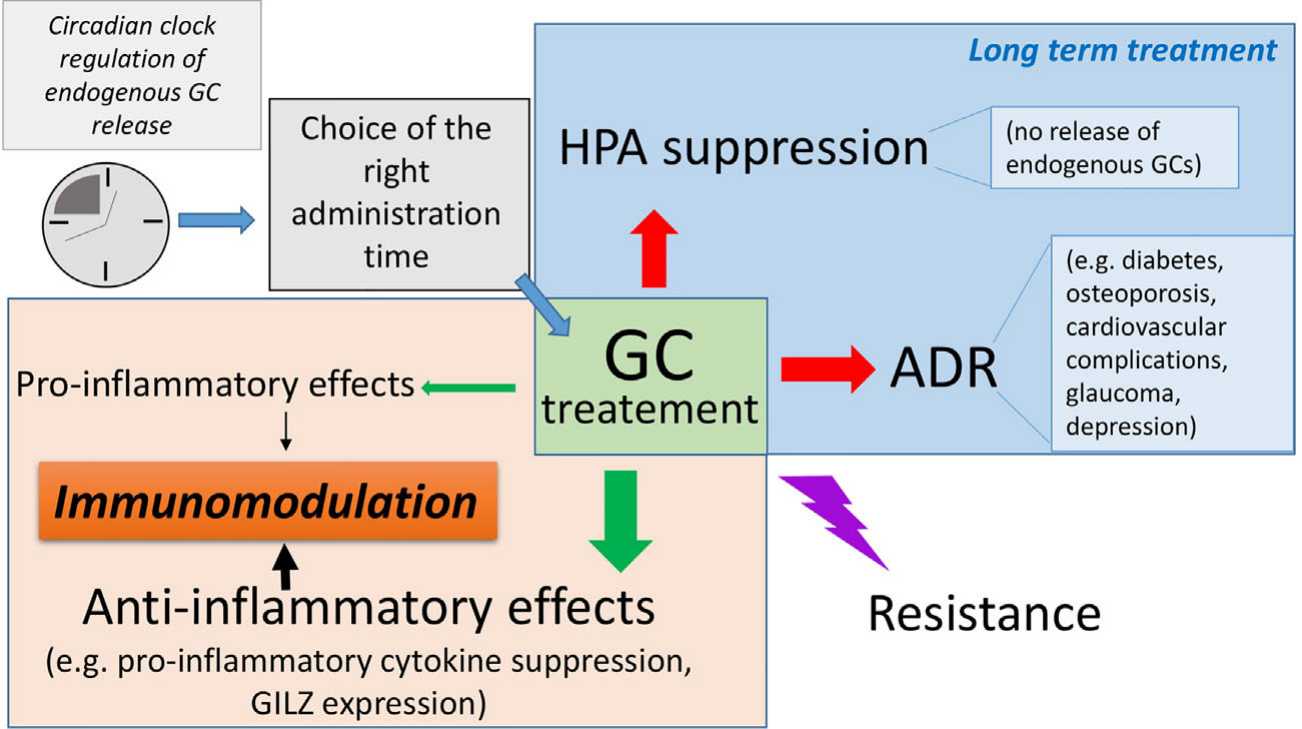
Complex regulation of glucocorticoid (GC) activity during pharmacological administration: GCs exert immunomodulatory activities, both by transactivating anti-inflammatory proteins such as glucocorticoid-induced leucine zipper (GILZ) and transrepressing pro-inflammatory genes, such as those encoding for pro-inflammatory cytokines, and therefore exerting powerful anti-inflammatory and immunosuppressive effects. They can also favor inflammation, e.g., by induction of inflammasome. Importantly, long-term GC treatment causes undesired adverse drug reactions (ADR), which can often result in severe pathologies such as diabetes, osteoporosis, and metabolic disorders. The hypothalamic–pituitary–adrenal (HPA) axis is inevitably suppressed after long-term treatment, thus indicating the use of dose-escalating regimens to avoid life-threatening consequences. The occurrence of resistance to GC efficacy can further complicate the management of autoimmune and inflammatory diseases, especially for diseases whose treatments other than GCs are not available.

Considering the multiple aspects related to GCs as drugs, the need to optimize GC therapy is crucial, especially in the case of rare diseases where GCs are the only choice and often used in combination with other drugs (**Figure 1**).

## Rare Pathologies That Require GC Treatment

Rare diseases are usually defined as those conditions that affect fewer than 200,000 people in the US and no more than 5 in 10,000 people within the general population in Europe. Independently of the numbers, a rare disease occurs in a small number of people compared to other diseases prevalent in the general population. Documented rare diseases range between 5,000 and 8,000, thus complicating the findings for appropriate pharmacological treatments, owing to the low number of cases for each pathology and its distribution across countries (36). The majority of rare diseases are life-threatening and genetic in origin, and most of them affect children, resulting in significant developmental problems or death. Therefore, treatments for rare diseases remain an unmet medical need. Any drug developed for the treatment of a rare disease is called “orphan drug.” Considerable costs associated with the development of these drugs often hinder the launch of a specific medical product in the market (37). Nevertheless, rare diseases whose pathogenesis has a proven inflammatory or autoimmune basis can be successfully treated with GCs, ensuring they are under control throughout patients’ lifetimes, although they cannot be cured. Needless to say, the severe adverse effects of long-term and high-dose GC therapy warrant the search of new pharmacological tools that could help avoid GC use while affording the same anti-inflammatory and immunosuppressive effects. A brief description of examples of GC treatments for some rare diseases follows.

Insulin autoimmune syndrome, a rare and systemic disease, is characterized by spontaneous episodes of hyperinsulinemic hypoglycemia caused by high titers of serum insulin autoantibodies. Self-remission occurs often in the affected individual; however, high-dose GC therapy is required and has resulted in successful outcomes because of its autoimmune origin. Other therapies are available, from immunosuppressive agents, such as azathioprine, to monoclonal antibodies-based therapies, such as rituximab (an anti-CD20 antibody) which targets IgG producer B cells (38).

Another systemic inflammatory orphan disease is relapsing polychondritis, which can be considered a syndrome, because it is frequently associated with other autoimmune conditions, including systemic vasculitis, RA, and spondyloarthritis. It is characterized by an inflammatory infiltrate, IgG deposits, and islands of cartilage with fibrosis. High-dose steroid therapy represents the first line of treatment only in life-threatening cases, followed by a switch to less toxic drugs in the maintenance phase, such as azathioprine. Non-steroidal anti-inflammatory drugs (NSAIDs) are also used in cases involving the nose, external ear, or joints. Biologicals including tocilizumab (an anti-IL-6 antibody), abatacept (a CTLA4 fusion protein), and TNF inhibitors can be used in patients who do not respond to standard therapies (39, 40).

Some rare diseases affect the skin, and sometimes, also other organs. Autoimmune bullous disorders encompass autoimmune diseases with high morbidity and mortality. Aberrant IgG and IgA autoantibody production directed against adhesion molecules of the epidermis or the dermal-epidermal basement membrane zone leads to a loss of skin adhesion. In pemphigus, IgG autoantibodies react against epidermal adhesion complexes of keratinocytes, whereas in pemphigoid diseases, loss of adhesion is typical of the basement membrane zone. The result of the action of these autoantibodies is the occurrence of mucosal or cutaneous blisters and erosions. First-line treatment involves systemic corticosteroids that may be combined with immunosuppressive agents, such as azathioprine, which help reduce the risk of relapse (41). As in other autoimmune diseases that involve autoantibody production by B cells, the anti-CD20 antibody rituximab has been found to be efficacious to such an extent that it has been recently considered as a first-line treatment (42).

The skin can be affected by pyoderma gangrenosum, a rare neutrophilic dermatosis with an inflammatory basis that is often associated with comorbidities such as IBD, arthritis, or malignancies. The pathogenesis of this condition remains still poorly understood, although an imbalance in the ratio of Treg/Th17 cells has been recently identified (43). Because of the absence of controlled and randomized clinical trials and the unknown pathogenic mechanisms, topical or systemic steroids have been used as first-line therapeutic agents, either alone (for example prednisone) or in combination with cyclosporin. However, the severity of adverse effects of these drugs has necessitated the identification of alternative agents, including anti-TNFα biologics (infliximab, adalimumab, golimumab, and etanercept) and IL-1 or IL-6 antagonists (anakinra and tocilizumab, respectively), whose beneficial effects need to be confirmed because of the paucity of available studies. A better understanding of the underlying molecular pathogenic pathways will allow more targeted and efficacious therapy (44).

Another rare disease that requires treatment with GCs is dermatomyositis, which can affect the skin, muscles and, occasionally, the lungs. The pathogenesis of this disease is still largely unknown, but inappropriate inflammatory mechanisms were found to be the basis of injuries in the skin or the parenchyma of muscles. Mild disease can be treated with topical GCs, whereas more severe diseases are usually treated with antimalarials as first-line treatment. However, oral systemic GCs coupled with immunosuppressants are generally used as therapy for both skin and muscle diseases. Other drugs include biologicals such as rituximab, abatacept, and tocilizumab (45).

The combination of GCs and rituximab was highly efficacious in increasing the response rate and prolonging the relapse-free survival phase in patients with warm-antibody reactive autoimmune hemolytic anemia, in comparison with that in the patients treated with prednisolone. This form of acquired hemolytic anemia is caused by the formation of autoantibodies directed against antigens of the red blood cell membrane, for which the recommended first-line treatment is prednisolone (46).

For life-threatening immunological syndromes such as hemophagocytic lymphohistiocytosis, GCs remain the best choice as first-line treatment. The pathogenesis of this condition, although largely unknown, involves sustained activation of the immune system, especially CD8+ cells and macrophages, and uncontrolled cytokine release, notably large amounts of IFNγ. Immunosuppressive and anti-inflammatory drugs are the only effective therapies, although newer drugs are being tested both in preclinical and clinical studies. Among these newer drugs, small molecule inhibitors of Janus kinases (JAKs) have proven to be efficacious because of the inhibition of IFNγ and IL-6 signals (47).

GCs represent the only choice for the treatment of IgG4-related disease, which is an immunoinflammatory disorder characterized by lymphoplasmacytic infiltrates of plasma cells bearing IgG4 on their surface. The disease mostly involves the pancreas, but other organs can be affected by infiltrates consisting of CD4+ T cells, B cells, and IgG4-plasma cells. Fibrosis, phlebitis, and eosinophilia are other pathological features of this disease (48). Monotherapy with either prednisolone or prednisone can induce remission in 82%–100% of patients when used as shortly as 3-4 weeks, followed by a tapering dosage. According to the Japanese guidelines (75% of patients are Japanese), a low-dose maintenance GC therapy is recommended for patients at high risk of relapse (49). The attempt to use biologics such as rituximab has proven unsuccessful because of the high percentages of relapse, but was efficacious in obtaining a good response in the totality of patients (50). IgG4-related hypophysitis is a recently identified subtype of hypophysitis that can be successfully treated with prednisone, which, occasionally, in case of relapse, can be continued at the maintenance dose for up to 3 years or even more (51). The same treatment schedule and dosage applies to other types of hypophysitis.

Although not in monotherapy, GCs are used in induction therapy for the treatment of anti-neutrophil circulating antibodies (ANCA)-associated vasculitis, a systemic, potentially life-threatening autoimmune disease that affects multiple organs and encompasses granulomatosis with polyangitis and microscopic polyangitis. Glomerulonephritis, which occurs frequently, can lead to renal failure; thus, prompt initiation of therapy is important to obtain rapid control of the disease. Cyclophosphamide is often used in combination with GCs, but the excessive immunosuppression and subsequent infections can result in high rates of mortality. Thus, there is a need for new therapies with both reduced toxicity and improved disease control (52–54).

Primary angiitis is a rare inflammatory disease of the central nervous system (CNS). Cells of the immune system infiltrate the CNS blood vessels, leading to thickening of the vessel walls and subsequent reduced circulation or, conversely, blood leakage and hemorrhage. Since the onset of this disease is sometimes insidious but progressive, prompt initiation of induction treatment with a combination of high-dose i.v. GCs and cyclophosphamide is important. This combination therapy can help avoid relapses, and this pharmacological approach is similar to the above-described orphan diseases. In addition, biological agents such as rituximab or infliximab have proven to be efficacious, but they represent an alternative option for patients who are intolerant to conventional therapies. After induction therapy, maintenance is achieved with corticosteroid-sparing low-risk immunosuppressants (azathioprine, mycophenolate mofetil, and metothrexate), with the aim of preventing severe side effects and relapses (55).

Although the majority of rare diseases do not show gender-specific patterns, some are more prevalent in females (in older patients). One such disease is juvenile localized scleroderma, a rare pediatric rheumatic condition associated with skin thickening and fibrosis that also occurs in adults. In this condition, inflammatory cells such as lymphocytes, eosinophils, and plasma cells infiltrate the reticular dermis, occasionally with the formation of edema. Anti-nuclear autoantibodies can be found in about 40% of patients. The recommended therapy includes GCs in combination with methotrexate, although there is no consensus on the dosage. Prednisone and prednisolone are used for oral therapy. The second-line therapy encompasses hydroxychloroquine, tocilizumab, infliximab, and abatacept (56).

**Figure 2:**
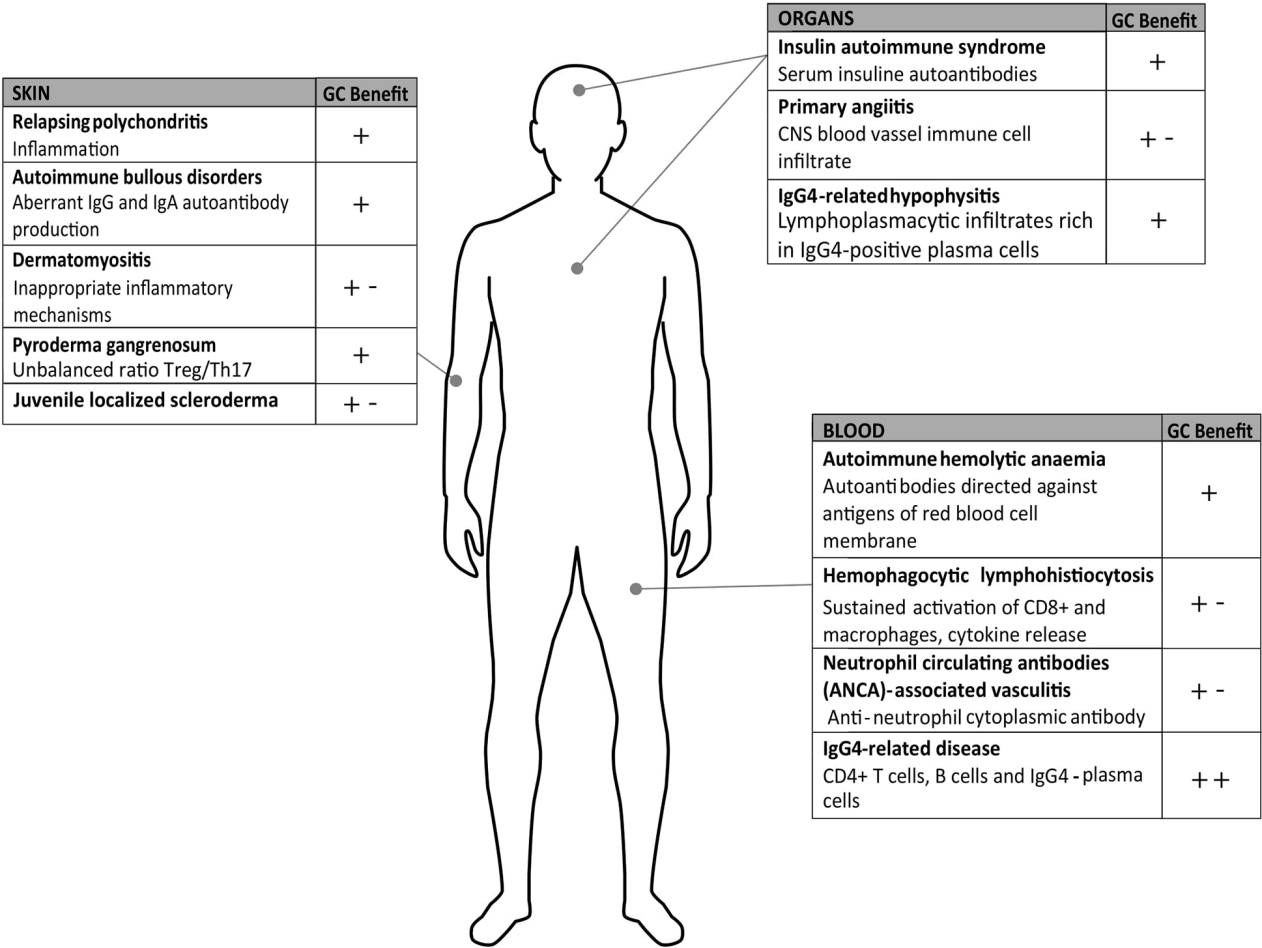
Rare diseases on autoimmune or inflammatory basis. The figure summarizes the pathologies described in the text and the known pathogenesis and highlights the glucocorticoids (GC) benefits as monotherapy (+) or combination therapies (+-); (++) benefit represents remission close to 100%.

For all the aforementioned diseases (**Figure 2**) and those not discussed in this review, when GC long-term therapy is needed, a careful risk-benefit assessment must be considered because of the severe adverse effects of GCs that often turn into comorbidities, including diabetes, infections, osteoporosis or even cardiovascular pathologies. Therefore, new strategies must be pursued with the aim of identifying new effective drugs.

## Discussion: GILZ-Based Proteins as Potential Pharmacological Tools

A review of literature clearly highlights the difficulty in establishing an efficacious treatment protocol for rare diseases regardless of the use of GCs. Several problems are linked to this aspect, starting from the paucity or complete absence of randomized, placebo-controlled clinical trials and the limited literature highlighting comparisons across therapies. As a result, for some rare pathologies, there is no established dose regimen for the use of GCs, which is based on clinical practice and sporadic or retrospective studies on small groups of patients (**Table 1**). In such cases, the treatment is tailored according to each clinical case, the patient’s response, and the time of the diagnosis in the progression of disease. Furthermore, the relative disinterest on the part of the biomedical industry to develop therapeutic agents for rare diseases is dictated by the small market potential, which, undoubtedly, leaves several clinical problems unsolved. Finally, from the economic point of view, care for a patient with a rare disease is much more expensive that for patients suffering from a common pathology. Rare diseases require a therapeutic challenge that can focus the efforts to optimize new pharmacological tools for their treatment. On the basis of what is known about the pathophysiology of autoimmune and inflammatory rare diseases, a field of research that is largely unexplored, new targets can be identified to allow the use of new drugs either in monotherapy or in combination. As an example, avacopan, a complement 5a receptor inhibitor, has proven to be effective in replacing high-dose GCs for the treatment of ANCA-associated vasculitis (77). Other experimental therapies include the use of monoclonal antibodies other than rituximab, but targeting the same B cells, such as ofatumumab, with optimized cytotoxicity, for phemphigus treatment. Since B cell intracellular signals activate p38MAPK, inhibitors of p38MAPK may represent an alternative strategy in pemphigus when applied topically, thereby avoiding the severe adverse events observed with oral administration (61). Other small-molecule inhibitors include tofacitinib, a Janus kinase (JAK)-1/3 inhibitor, and ruxolitinib, a selective JAK-1/2 inhibitor, which suppress interleukin and interferon signaling in dermatomyositis (45). Although they have been used in a small number of patients, they proved promising. Therefore, several efforts are needed to overcome the numerous problems linked to the treatment of rare diseases, even though these new drugs may occasionally show dose-limiting effects. We propose the potential use of a recombinant protein that was found to be therapeutic in some experimental models of autoimmune diseases in preclinical studies. The TAT-GILZ recombinant molecule is a fusion protein of full-length GILZ, an early GC-induced protein that mimics several beneficial effects of GCs without exerting GC-related adverse effects (13, 78). The first attempt in the use of TAT-GILZ was performed in a mouse model of DNBS-induced colitis, in which TAT-GILZ treatment successfully reduced the severity of spontaneously developed colitis in IL-10-knock-out mice. GILZ can inhibit NF-κB, a pivotal transcription factor in the regulation of pro-inflammatory cytokines, thereby contributing to the regulation of the CD4+ response in the gut (79). In the same model of colitis, but in a different strain of genetically modified mice, the *GILZ* B cell-conditional knock-out mice (GILZ B cKO), 3 day-treatment with the recombinant TAT-GILZ protein reversed the symptoms of DNBS-induced colitis, similar to wild-type mice (80, 81). In the context of a neutrophilic inflammation in a model of LPS-induced pleurisy in mice, TAT-GILZ was able to lead to inflammation resolution by decreasing cytokine production and promoting apoptosis in neutrophils (82). Another type of GILZ peptide was employed in the experimental encephalomyelitis, a mouse model of human multiple sclerosis. In this model, the proline-rich portion of the carboxyl terminus of GILZ protein (GILZ-P), which can bind the p65 subunit of NF-κB, was found to inhibit the transactivation of inflammatory cytokines, thus ameliorating the disease (83). Another interesting study demonstrated that TAT-GILZ can reduce Th-17 frequency and increase Treg cells in another inflammatory context, such as acute kidney injury (84). Furthermore, in human B lymphocytes, intracellular delivery of the HHph-GILZ peptide inhibited cell proliferation *in vitro*, explaining the observation of reduced GILZ in B cells of *systemic lupus erythematosus* patients and *lupus*-prone mice (85). Another proof of the efficacy of GILZ-based peptides is given by the use of a synthetic TAT-GILZ peptide (GILZ-p) in a model of ocular uveitis in rats. The inflammatory response was counteracted by the intravitreal injection of GILZ-p, which could reduce the expression of IL-1β and TNF-α (86).

**Table 1:** Summary of the presented rare diseases, related treatments and references.

Disease	Treatment	References
**Insulin autoimmune syndrome**	Prednisone (first line), azathioprine, acarbose, thiamazole, biologics (anti-CD20 -*rituximab*-)	(38, 57)
**Relapsing polychondritis**	Steroids (first line), azathioprine, Non-steroidal anti-inflammatory drugs (NSAIDs), biologics (anti-TNFα -*infliximab, adalimumab, golimumab, certolizumab, etanercept-*, -anti-IL-6 -*tocilizumab*-, anti-IL-1 -*anakinra-*, CTLA-4 Ig -*abatacept*-, *rituximab*)	(58, 59)
**Autoimmune bullous disorders, pemphigus, pemphingoid diseases**	Prednisone (first or second line), clobetasol propionate, mycophenolate mofetil, methotrexate, azathioprine, combination of tetracyclines, doxycycline or minocycline and niacinamide, IVIG, biologics (*rituximab*, anti-IgE -*omalizumab*-).	(41, 60–62)
**Pyoderma gangrenosum**	Prednisone/prednisolone (first line), tacrolimus, sodium cromoglycate, cyclosporine, methotrexate, mycophenolate mofetil, azathioprine, intravenous immunoglobulins, (IVIG), biologics (*infliximab*, *adalimumab, golimumab, etanercept*, anti-IL-12 and anti-IL-23 –*ustekinumab*-, *anakinra*, anti-IL1β -*canakinumab*-, *tocilizumab*), JAK/STAT inhibitors (tofacitinb),	(44, 63)
**Dermatomyositis**	Steroids (first or second line), antimalarials, methotrexate, mycophenolate mofetil, IVIG, biologics (*rituximab, abatacept, tocilizumab*), tofacitinib	(45, 64)
**Warm Antibody reactive autoimmune hemolytic anemia**	Prednisolone (first line), azathioprine, biologics (*rituximab*)	(46, 65–67)
**Haemophagocytic limphohistiocytosis**	Methylprednisolone/dexamethasone/hydrocortisone (first line), biologics (*rituximab, anakinra*, anti-CD52 *-alemtuzumab-*), etoposide, cyclosporine A, methotrexate	(47, 68)
**IgG4-related disease**	Prednisolone/prednisone (first line), azathioprine, 6-mercaptopurine, methotrexate, mycophenolate mofetil, biologics (*rituximab*, *infliximab*), hydroxychloroquine	(48, 50, 69, 70)
**IgG4-related hypophysitis**	Prednisone (first line), methotrexate, biologics (*rituximab*)	(51, 71, 72)
**Neutrophil circulating antibodies (ANCA)-associated vasculitis**	Prednisone (first line), cyclophosphamide, methotrexate, mycophenolate mofetil, biologics (*tocilizumab*)	(52, 53, 73)
**Primary angiitis**	Prednisone (first line), cyclophosphamide, methotrexate, mycophenolate mofetil, azathioprine, IVIG, biologics (*rituximab, infliximab, etarnecept*)	(55, 74, 75)
**Juvenile localized scleroderma**	Prednisone/prednisolone (first line), methotrexate, hidroxychloroquine, imiquimod, cyclosporine A, biologics (*tocilizumab, infliximab, abatacept*), tofacitinib	(56, 76)

Management of the listed pathologies can be found in the indicated references.

Since GILZ is a pivotal intermediate of GC anti-inflammatory and immunoregulatory actions, the rationale for the use of GILZ protein-based pharmaceuticals is attractive in the rare diseases described above. Several autoimmune and inflammatory rare diseases share common altered immune responses, including uncontrolled B cell activation (see insulin autoimmune syndrome, autoimmune bullous disease, etc.). B cells are targets of GILZ effects, since GILZ is indispensable to control the over production of IFNγ in B cells, as demonstrated by the elevated levels of IFNγ in GILZ B cKO B lymphocytes. These mice are prone to develop a severe disease in the experimental model of colitis, in which IFNγ-secreting B cells have a pathogenic role (80). Moreover, GILZ-deficient mice develop a progressive B lymphocytosis, with expansion of B220+ cells in the bone marrow because of an increased survival of B cells (87). GILZ plays a role not only in B cells but also in T cell subtypes. A study demonstrated that GILZ is involved in the regulation of Th17 activity, in that it maintains a threshold for Th17 activation in an experimental model of psoriasis. Interestingly, GILZ expression inversely correlates with disease severity, suggesting that low amounts of GILZ may worsen this disease and/or may be part of the pathogenesis (88–90). Moreover, GILZ-deficient mice show spontaneous production of IL-17A and IL-22 in the imiquimod model of psoriasis, and their dendritic cells produce high amounts of IL-1, IL-23, and IL-6 (90). A recent study in pyoderma gangrenosum reported that, in the skin of patients suffering from this disease, Th17 cells are increased in number whereas Treg cells are reduced, with their balance shifted toward the Th17 pathogenic cells (43). The potential treatment with a GILZ protein would prove beneficial to restore the right balance of Th17/Treg cells, since GILZ is also involved in the production of Treg cells, as demonstrated by GILZ conditional knock-out mice (91). In addition, because pyoderma gangrenosum belongs to those rare inflammatory diseases referred to as neutrophilic dermatosis, GILZ treatment could reduce neutrophil activation, since we have previously demonstrated that GILZ is indispensable to restrain the activity of neutrophils in the site of inflammation (44, 92).

**Figure 3:**
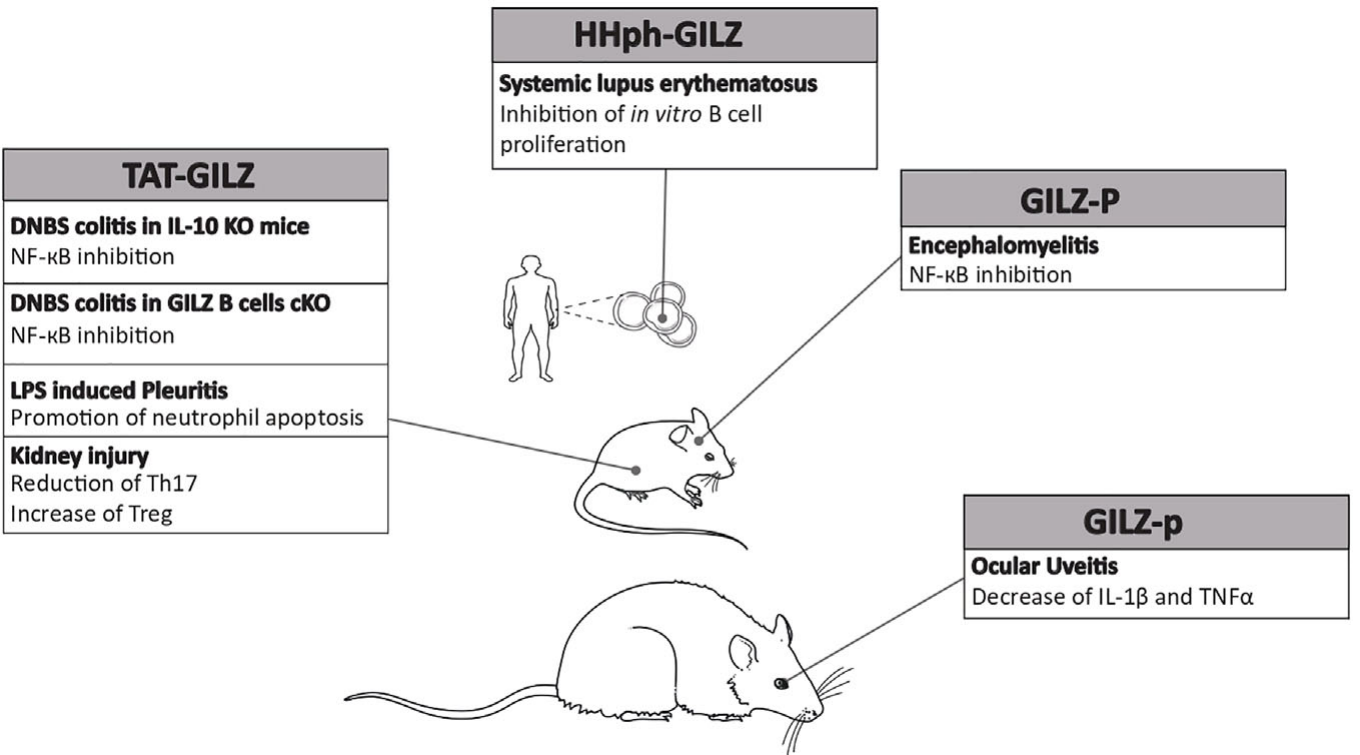
Efficacy of glucocorticoid-induced leucine zipper (GILZ)-based proteins in experimental models. Summary of GILZ proteins/peptides that have been used in rodent models of autoimmune or inflammatory diseases and proven efficacious. The underlying molecular and cellular mechanisms are also indicated.

Finally, in all rare diseases characterized by an overproduction of pro-inflammatory cytokines, GILZ might be the protein that counteracts the increase. GILZ was found to inhibit the pro-inflammatory effects of TNFα in human adipocytes, to reduce macrophage inflammatory protein 1 (MIP-1) in macrophages, to inhibit the expression of the adhesion molecules like ICAM-1 in endothelial cells, and to interfere with other inflammatory mechanisms (93–96). More importantly, GILZ can heterodimerize with NF-κB, thus inhibiting the transactivation of downstream known pro-inflammatory genes. Therefore, GILZ, as a regulator of uncontrolled immune response, might be an ideal candidate for the development of new biological drugs (**Figures 2** and **3**). Up to date there are still no trials in humans, because further pre-clinical studies about the pharmacokinetics and pharmacodynamics of exogenous GILZ protein, as well as about its possible toxicity, are needed. It is reasonable to hypothesize a future use for the treatment of rare inflammatory and autoimmune diseases with a high benefit/cost ratio, since protein-based therapies are expensive but need low doses and less frequent dosing regimens. Furthermore, no comorbidities are expected to develop with no need of additional pharmacological treatments. Overall, GILZ-based proteins may represent an actual alternative to GCs.

## Conclusions

Rare diseases with an inflammatory basis can be successfully treated with GCs, but the consequences of long-term or high-dose treatments can be detrimental. Thus, there is an urgent need to discover new drugs either to reduce the GC doses in co-treatments or hopefully replace them. Knowledge of the pathophysiology of these diseases is mandatory, so that new targets can be identified. On the other hand, proteins like GILZ, which mimic the GC beneficial effects, could be ideal candidates to reduce inflammation where its components are causative or contribute to the pathogenesis of the pathology. Furthermore, the ability of GILZ to change the balance between immune cells toward an anti-inflammatory or autoimmune phenotype can be exploited to restore a correct immune response. GILZ-based proteins may represent the next step in the treatment of rare inflammatory diseases, predicting their future hope and use in humans.

## Author Contributions

All authors contributed to the article and approved the submitted version.

## Funding

This work was supported by a grant from the Italian Ministry of Education, Universities and Research to SR (PRIN 2017B9NCSX).

## Conflict of Interest

The authors declare that the research was conducted in the absence of any commercial or financial relationships that could be construed as a potential conflict of interest.
